# Clinical and ethical perspectives of ovarian stimulation and oocyte cryopreservation in adolescents: 6 years experience from a tertiary centre

**DOI:** 10.1093/hropen/hoaf005

**Published:** 2025-01-24

**Authors:** Sania Latif, Melanie Davies, Emily Vaughan, Dimitrios Mavrelos, Stuart Lavery, Ephia Yasmin

**Affiliations:** Institute for Women’s Health, University College London, London, UK; Reproductive Medicine Unit, University College London Hospital, London, UK; Institute for Women’s Health, University College London, London, UK; Reproductive Medicine Unit, University College London Hospital, London, UK; Reproductive Medicine Unit, University College London Hospital, London, UK; Institute for Women’s Health, University College London, London, UK; Reproductive Medicine Unit, University College London Hospital, London, UK; Institute for Women’s Health, University College London, London, UK; Reproductive Medicine Unit, University College London Hospital, London, UK; Institute for Women’s Health, University College London, London, UK; Reproductive Medicine Unit, University College London Hospital, London, UK

**Keywords:** fertility preservation, adolescence, oocyte cryopreservation, ovarian stimulation, egg freezing, cryopreservation, female infertility, oocyte quality, egg freezing, ovary

## Abstract

**STUDY QUESTION:**

What are the clinical and ethical challenges of performing ovarian stimulation and oocyte cryopreservation in adolescents and the barriers to providing treatment?

**SUMMARY ANSWER:**

Our study shows that, in one of the largest case series to date in this population, post-pubertal adolescents as young as age 13 years can undergo ovarian stimulation and oocyte cryopreservation with a response comparable to adults.

**WHAT IS KNOWN ALREADY:**

Fertility preservation in adolescents has not been well studied, with little data available in the existing literature. Referrals for fertility preservation in adolescents are increasing due to developments in childhood cancer treatments, which have led to a growing population of children at risk of developing premature ovarian insufficiency. Those with certain benign conditions or gender incongruence also face this challenge. All established fertility preservation guidelines state that where there is a risk to fertility, oocyte cryopreservation should be offered to post-pubertal females. However, counselling and consenting young people about fertility decisions is an ethically complex area, and assessing capacity to consent in this age group is not straightforward.

**STUDY DESIGN, SIZE, DURATION:**

This was a retrospective observational cohort study of 182 referrals for fertility preservation counselling to a specialist unit, and we present outcomes for the 33 adolescents who underwent 36 cycles of ovarian stimulation and oocyte cryopreservation between January 2018 and January 2024.

**PARTICIPANTS/MATERIALS, SETTING, METHODS:**

We included patients aged 13–18 years who underwent ovarian stimulation and oocyte cryopreservation for fertility preservation due to high or intermediate risk of gonadotoxicity from medical or surgical treatment at a public-funded specialist unit. The primary outcome was oocyte yield; secondary outcomes included oocyte maturity rate, complications, and dropout rate. Data were retrieved from a prospectively managed database.

**MAIN RESULTS AND THE ROLE OF CHANCE:**

There was a total of 182 referrals received, and of these, 33 patients underwent 36 cycles of ovarian stimulation and oocyte cryopreservation. Indications for fertility preservation included malignancy *n* = 19/36 (54%), ovarian cyst surgery *n* = 7/36 (19%), immunological disorders *n* = 4/36 (11%), benign haematological disease *n* = 2/36 (6%), gender reassignment treatment *n* = 3/36 (8%), and genetic conditions *n* = 1/36 (3%). The youngest child who underwent ovarian stimulation was aged 13 years and 10 months at the time of egg collection; the minimum time from menarche to ovarian stimulation was 4 months, the median AMH (anti-Müllerian hormone) was 16.7 pmol/l (range 2.8–36.9 pmol/l), and the antral follicle count (AFC) was 11 (3–36). The median number of cryopreserved oocytes was 14 (range 4–39), and the oocyte maturity rate was 85% (35–100%). Ultrasound monitoring was performed transabdominally in 5/33 (15%) and transvaginally in 28/33 (85%). Egg collection was performed transvaginally in all cases in this cohort. All cycles proceeded to completion. All adolescents were counselled in association with a family member to obtain informed consent, and all were assessed as able to comprehend discussions.

**LIMITATIONS, REASONS FOR CAUTION:**

In view of concerns regarding increased aneuploidy rates in this age group compared to women in their twenties, there is a need for long-term outcome studies expanding on our findings with data on livebirths to support clinicians needing to counsel patients and perform oocyte cryopreservation in adolescents.

**WIDER IMPLICATIONS OF THE FINDINGS:**

Clinician experience, correct setting, and availability of funding will enable a permissive environment for oocyte cryopreservation in adolescents. In our experience, transvaginal egg collection is an accepted procedure when counselled appropriately.

**STUDY FUNDING/COMPETING INTEREST(S):**

No funding was received for this work. No competing interests are declared.

**TRIAL REGISTRATION NUMBER:**

N/A.

WHAT DOES THIS MEAN FOR PATIENTS? This study explored clinical and ethical factors for consideration when performing egg freezing in teenagers.

The need to perform egg freezing in teenagers is increasing due to both improved survival rates in children following cancer treatments and also an increase in the number of non-cancerous conditions requiring medical or surgical treatment, which can affect fertility, including transgender care.

Egg freezing in teenagers has not yet been well studied. This study shows that egg freezing in teenagers can be performed successfully when undertaken in the correct setting by an experienced team and requires appropriate counselling. There is still a need for further studies looking at live birth rates using frozen eggs from teenagers.

## Introduction

The need for performing fertility preservation in adolescents is increasing because, alongside recent improvements in childhood cancer survival, there has also been an increase in transgender care and in benign conditions requiring gonadotoxic treatment, such as haemoglobinopathies and immunological disorders requiring stem cell transplant. This has led to a higher representation of adolescents in fertility preservation clinics (Mulder *et al.*, 2021; Slonim *et al.*, 2023; The Tavistock and Portman NHS Foundation Trust, 2023). Specific cancer diagnoses arise more commonly during adolescence, including sarcomas and neurological and haematological malignancies (National Cancer Registration and Analysis Service, 2019). There is also the first appearance of specific benign conditions during adolescence, such as vasculitis and certain renal diseases requiring treatment with gonadotoxic agents (Sönmez *et al.*, 2019; Nair *et al.*, 2023). Other groups with threat to fertility at this age include those with gender incongruence undergoing gender-affirming treatment and genetic mutations predisposing to high risk of premature ovarian insufficiency, such as fragile X syndrome and Turner syndrome (Yasmin *et al.*, 2018).

Adolescence is a time of substantial physical, emotional, and psychological changes and is characterized by maturation of the hypothalamo–pituitary–ovarian (HPO) axis, which can take several years to become established (Yasmin *et al.*, 2021). Performing fertility preservation during this developmental transition carries increased complexities—both physical, dependent upon pubertal stage and medical diagnosis, and psychological, in relation to acceptance of a new diagnosis, processing information, and decision-making (Logan *et al.*, 2019; Mertes and Pennings 2022). Future infertility can be a significant source of stress and anxiety for adolescents and their parents, and illness during this time requires special care from healthcare professionals (Nilsson *et al.*, 2014).

Ovarian stimulation and oocyte cryopreservation are proven forms of fertility preservation in adults, though they have not been well studied in adolescents (Anderson *et al.*, 2015; Cobo *et al.*, 2016; Oktay *et al.*, 2018; Anderson *et al.*, 2020). Uncertainties remain regarding the response of an adolescent ovary to ovarian stimulation, reliability of ovarian reserve markers as predictors of response to ovarian stimulation, and the ability of young individuals to sustain the psychological burdens of fertility preservation procedures (Anderson *et al.*, 2022; Lavery *et al.*, 2016; McDougall *et al*., 2018; Garg *et al.*, 2019). There are also questions surrounding oocyte quality at a young age, with studies of follicle morphology demonstrating an increase in abnormal types (Anderson *et al.*, 2014). Counselling and consenting young people about fertility decisions, often alongside giving a life-changing diagnosis, is an ethically complex area, and assessing capacity to consent in children is not straightforward (McDougall *et al.*, 2018; Young *et al.*, 2019; Mertes and Pennings, 2022).

The existing literature on performing oocyte cryopreservation in adolescents consists largely of case reports and case series (Oktay *et al.*, 2010; Reichman *et al.*, 2012; Oktay and Bedoschi, 2014; Lavery *et al.*, 2016; Chen *et al.*, 2018; Peddie and Maheshwari, 2018; Garg *et al.*, 2019; Hipp *et al.*, 2019; Rodriguez-Wallberg *et al.*, 2019; Amir *et al.*, 2020; Manuel *et al.*, 2020; Insogna *et al.*, 2020; Martin *et al.*, 2021; Barrett *et al.*, 2022; Martel *et al.*, 2022). A recent systematic review suggested that there is a need for larger studies looking at clinical outcomes of performing oocyte cryopreservation in adolescents so that clinicians can counsel young people and their families appropriately (Slonim *et al.*, 2023). We present clinical outcomes of performing ovarian stimulation and oocyte cryopreservation for fertility preservation in adolescents aged 13–18 years. Our study generates one of the largest adolescent cohorts to date, highlighting clinical and ethical considerations and the barriers to provision identified from performing 36 cycles of oocyte cryopreservation.

## Materials and methods

This was a retrospective, observational cohort study of adolescents aged 13–18 years undergoing ovarian stimulation and oocyte cryopreservation between January 2018 and January 2024 at a specialist unit for fertility preservation funded by the UK National Health Service (NHS). Data were retrieved from a prospectively managed database. The primary outcome was oocyte yield; secondary outcomes included oocyte maturity rate, complications, and dropout rate. We noted demographic data, ovarian reserve, route of ultrasound monitoring, response to ovarian stimulation, and route of oocyte retrieval. Documentation on counselling conversations and assessment of psychological maturity was retrieved from clinical records.

### Clinical selection criteria

#### Inclusion criteria

The following criteria were applied for offering oocyte cryopreservation in accordance with ESHRE guidance on female fertility preservation (Anderson *et al.*, 2020).

- Post-menarchal status
- Intermediate to high risk of gonadotoxicity from medical or surgical treatment
- Medical fitness for the procedure
- Oocyte cryopreservation benefits outweigh the risks
- Capacity to comprehend treatment and provide consent. *(As per the Human Fertilization and Embryology Authority Code of Practice, the individual’s consent is required for gamete storage, with a need to assess the capacity to consent for ovarian stimulation, egg collection, and gamete storage, as well as a signature by their representative.)*
- Funding availability

#### Exclusion criteria

- Pre-menarchal status
- Medically unfit for the procedure
- Insufficient time to complete oocyte cryopreservation
- Low risk of gonadotoxicity from medical or surgical treatment
- Lack of capacity to comprehend treatment and provide consent

### Pre-procedural assessments

#### Ovarian reserve

Ovarian reserve was measured according to routine practice using AMH (anti-Müllerian hormone) concentration and antral follicle count (AFC), obtained by performing a transvaginal or transabdominal ultrasound. We recorded concomitant use of hormonal treatment such as the contraceptive pill, progestins for menstrual suppression, and GnRH analogues due to their influence on ovarian reserve tests.

#### Medical fitness

Where appropriate, anaesthetic assessment was performed pre-operatively to assess medical fitness for ovarian stimulation and egg collection, for example, those with medical co-morbidities which pose a risk to deep sedation during egg collection, such as lymphoma patients with large mediastinal masses.

#### Consent

Patients received an assessment for capacity to consent performed by a reproductive medicine specialist with expertise in the field of paediatric and adolescent gynaecology, trained to facilitate decision-making in children and their families. The concept of Gillick competence was applied, in which children aged 16 and over are entitled to consent to their own treatment, and children aged 16 and under can consent to their own treatment if they are believed to have enough intelligence, competence, and understanding to fully appreciate what will be involved in their treatment (National Health Service Consent to Treatment, 2024). Young individuals were seen alone as well as with their parents. A second consultation was provided where needed, and counselling support was provided by a psychologist. All patients received a follow-up call from the clinical nurse specialist enquiring about their physical and psychological well-being, although a formal psychological assessment was not performed. A telephone translation service (Language Line) was available for individuals who did not speak English (*n* = 2/33, 6%).

### Monitoring

Pre-procedural discussion included route of ultrasound monitoring (transvaginal or transabdominal) and route of egg collection (transvaginal or transabdominal) to highlight the merits of transvaginal egg collection. The route of ultrasound monitoring was determined based on patient choice, and transvaginal ultrasound was offered to those patients who had previously had vaginal intercourse or used tampons. Counselling was delivered by experienced clinicians trained in the field of paediatric and adolescent gynaecology, who had completed RCOG (Royal College of Obstetricians and Gynaecologists) accredited sub-specialty training in reproductive medicine, including specialist training in the management of complex pathology in paediatric and adolescent gynaecology and had practised at consultant level for 10 years. These clinicians had received consistently positive feedback on their counselling skills from patients and colleagues via formal online assessment (www.sardjv.co.uk).

### Ovarian stimulation protocol

Two types of stimulation protocols were used; either the antagonist protocol or progestin-primed ovarian stimulation. Patients were initiated on human menopausal gonadotropin (Menopur; Ferring, Kiel, Germany) in combination with recombinant FSH (Bemfola; Gedeon Richter, Budapest, Hungary) at a dose determined by ovarian reserve tests. In antagonist cycles, GnRH antagonist (Cetrotide, 0.25 mg OD; Merck, Darmstadt, Germany) was initiated once the lead follicle had attained a mean diameter of 12 mm. In progestin-primed cycles, progestin (Provera, 10 mg BD; Pfizer, Ascoli Piceno, Italy) was initiated on the fourth day of gonadotrophin administration. An aromatase inhibitor (Letrozole, 2.5 mg OD; Novartis, Dublin, Ireland) was used in those undergoing fertility preservation for gender reassignment. Follicular development was assessed via transabdominal or transvaginal sonography in combination with serial measurement of serum estradiol concentrations. Trigger was administered when three lead follicles were >17 mm with a GnRH agonist (Buserelin; Neon Healthcare Ltd, Frankfurt, Germany). In those with pituitary suppression and low risk of ovarian hyperstimulation syndrome (OHSS), recombinant HCG (Gonasi, 10 000 IU; IBSA, Watford, UK) trigger was administered instead of GnRH agonist. Oocyte retrieval was performed 37 h following the trigger, and oocytes were vitrified. In dual stimulation cycles, ovarian stimulation was initiated 5 days following oocyte retrieval.

### Ethical approval

The purpose of this retrospective analysis was to improve service provision; therefore, approval was gained from the Women’s Health Divisional Clinical Director, and national ethics committee approval was not required.

## Results

**Figure 1.**
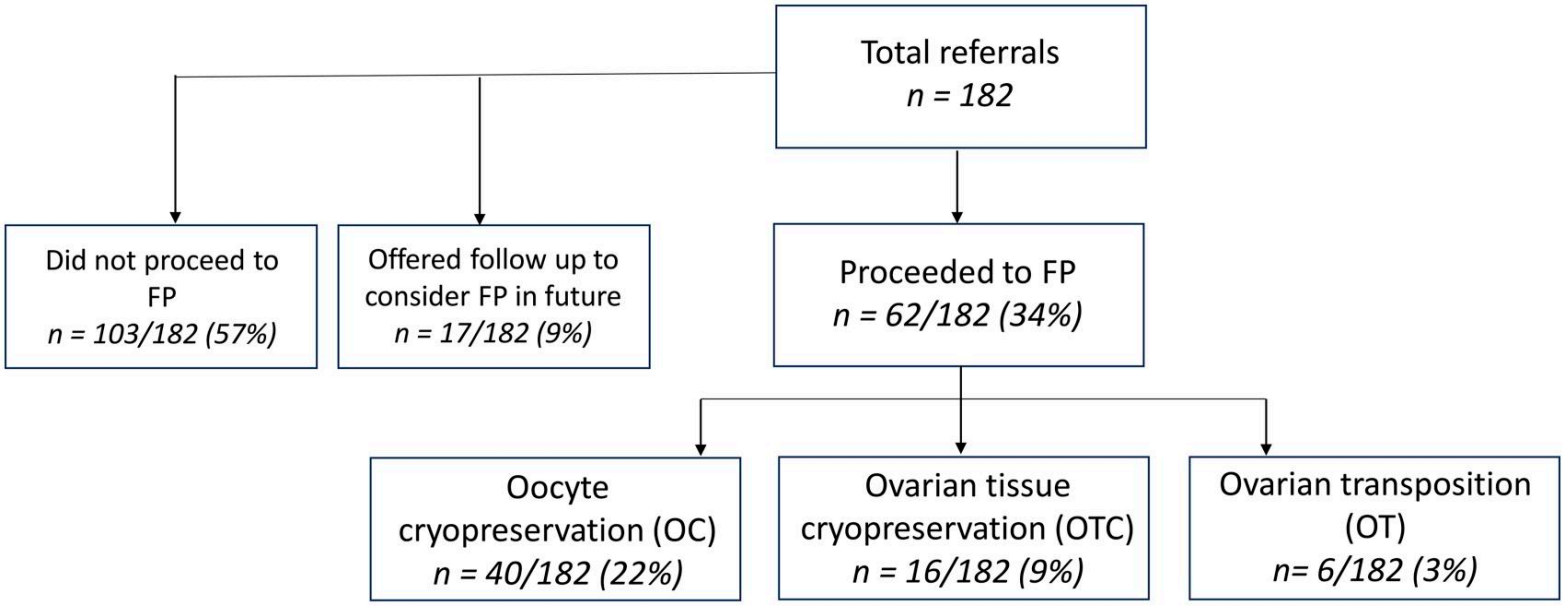
**Flow chart of referrals received for fertility preservation counselling in children aged 13–18 years between January 2018 and January 2024.** Of the 182 adolescents who received fertility counselling, 62/182 (34%) proceeded to a form of fertility preservation, and 120/182 (63%) did not. Of the 40 adolescents who underwent oocyte cryopreservation, 36 cycles were performed at our unit, and 4 were performed elsewhere due to NHS funding.

**Figure 2.**
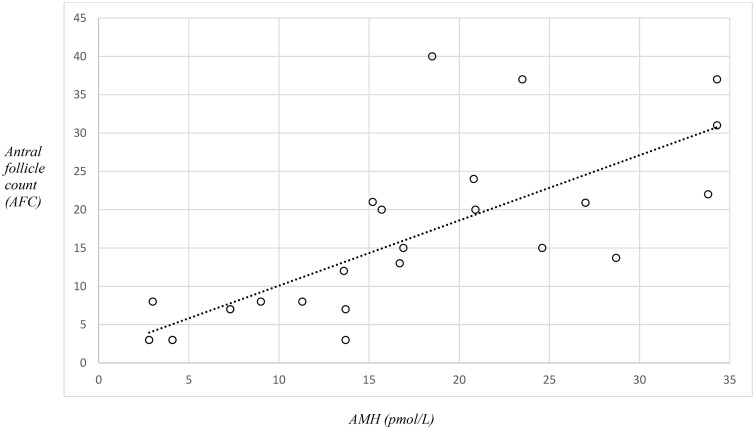
**Scatter plot showing correlation between anti-Müllerian hormone (AMH) and antral follicle count (AFC) in children aged 13–18 years.** The median AMH was 16.7 (2.8–36.9) pmol/l, and the median antral follicle count (AFC) was 11 (range 3–36).

**Figure 3.**
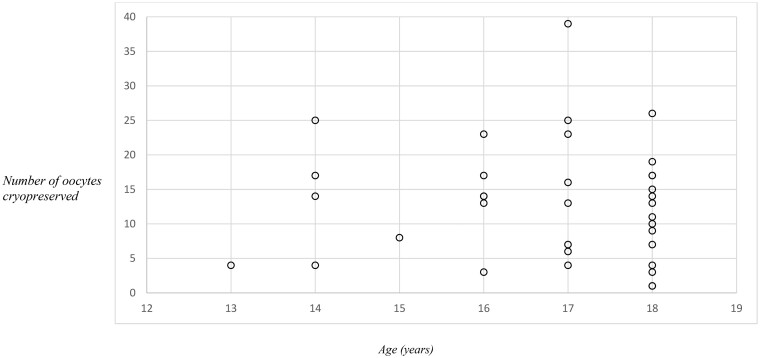
**Number of oocytes cryopreserved in children aged 13–18 years by age.** Response to ovarian stimulation in adolescents is comparable to that in adults, with an even spread in oocyte yield across the 13–18 years age group.

There was a total of 182 referrals received for fertility preservation counselling in children aged 13–18 years between January 2018 and January 2024 (Fig. 1). Overall, of the 182 adolescents who received fertility counselling, 62/182 (34%) proceeded to a form of fertility preservation, 17/182 (9%), and 103/182 (57%) did not proceed to a form of fertility preservation (Fig. 1). In this study, we present data on the 33 children who underwent 36 cycles of ovarian stimulation and oocyte cryopreservation. Indications for oocyte cryopreservation included malignancy 19/36 (54%), immunological disorders 4/36 (11%), gender reassignment treatment 3/36 (8%), ovarian cyst surgery 7/36 (19%), benign haematological disease 2/36 (6%), and Turner syndrome 1/36 (3%) (Table 1). All children were post-menarchal, with a median age of 16.6 years (range 13.8–18.6) and a minimum time from menarche to ovarian stimulation of 4 months. There were seven children who had previously received mild chemotherapy (ABVD—adriamycin/bleomycin/vinblastine/dacarbazine, CHOP—cyclophosphamide/doxorubicin/vincristine/prednisolone, FLAG-Ida—fludarabine/cytarabine/granulocyte-stimulating factor/idarubicin, Vincristine) and were subsequently planned for stem cell transplant. The median AMH was 16.7 (2.8–36.9) pmol/l, and the median AFC was 11 (range 3–36) (Fig. 2). Anytime-start was performed in 23/36 (64%) and early follicular phase start in 13/36 (36%). There were four children who underwent a dual stimulation cycle. In this cohort, gonadotropin stimulation was well tolerated by all patients. The median number of oocytes cryopreserved was 14 (range 3–39), the median number of metaphase II (MII) oocytes was 12 (3–22), and the oocyte maturity rate was 85% (35–100%) (Table 2, Fig. 3). Ultrasound monitoring was performed transabdominally in 5/33 (15%) children and transvaginally in 28/33 (85%). Egg collection was performed transvaginally in all cases in this cohort, comprising children from a diverse range of ethnicities and cultural backgrounds (see Table 1). All initiated cycles proceeded to completion. There was one case of moderate OHSS. There were no serious complications in any child. All children were counselled in association with a family member to obtain informed consent, and all were assessed as able to comprehend discussions. The child and parents were in agreement regarding their decision for fertility preservation in all cases. Reasons for not proceeding with fertility preservation are outlined in Table 3.

**Table 1.** Clinical characteristics of patients undergoing oocyte cryopreservation.

**Age (years)**	16.6 (range 13.8–18.6)
**BMI (kg/m^2^)**	22 (range 19–35)
**Ethnicity**	
White	16/33 (44%)
Asian	6/33 (18%)
Black	2/33 (6%)
Mixed	4/33 (12%)
Other	4/33 (12%)
Not disclosed	1/33 (3%)
**AMH (pmol/l)**	16.7 (range 3.0–36.9)
**AFC**	11 (range 3–36)
**Post-menarcheal**	
Yes	33/33 (100%)
No	0/33 (0%)
**Menarche (age in years)**	12 (range 11–15)
**Time from menarche to start of ovarian stimulation (months)**	54 (range 4–72)
**Indication for fertility preservation**	
Haematological malignancy (AML, ALL, Hodgkin’s lymphoma)	14/36 (39%)
Sarcoma	2/36 (6%)
Head and neck malignancy (nasopharyngeal carcinoma)	1/36 (3%)
Neurological malignancy (glioblastoma)	1/36 (3%)
Breast malignancy	1/36 (3%)
Ovarian cyst surgery (dermoid cyst, Gorlin syndrome, immature teratoma)	7/36 (19%)
Benign haematological disease	2/36 (6%)
Gender reassignment treatment	3/36 (8%)
Immune and metabolic disorders (DOCK8 deficiency, APDS2 deficiency, StAR type 2 CAH, chronic granulomatous disease)	4/36 (11%)
Turner syndrome	1/36 (3%)

AMH, anti-Müllerian hormone pmol/l; AFC, antral follicle count; AML, acute myeloid leukaemia; ALL, acute lymphoblastic leukaemia; APDS2, activated phosphoinositide 3-kinase delta syndrome; StAR type 2 CAH, steroidogenic acute regulatory protein type 2 congenital adrenal hyperplasia.

**Table 2.** Clinical outcomes for oocyte cryopreservation cycles.

	*n* (%)
**Ovarian stimulation protocol**	
Antagonist	26/36 (72%)
Progestin-primed ovarian stimulation	10/36 (28%)
**Timing of start**	
Any-time start	23/36 (64%)
Early follicular	13/36 (36%)
**Number of stimulation cycles**	
Single cycle	32/36 (89%)
Dual stimulation	4/36 (11%)
**Duration of stimulation (days)**	11 (range 8–15)
**Total dose of gonadotropins (IU)**	3000 (range 1350–5350)
**Peak estradiol (pmol/l)**	7585 (range 1743–25297)
**Number of oocytes retrieved**	14 (range 2–48)
**Percentage of metaphase II oocytes retrieved (%)**	85 (range 35–100%)
**Route of ultrasound scan monitoring**	
Vaginal	31/36 (86%)
Abdominal	5/36 (14%)
**Route of egg collection**	
Vaginal	36/36 (100%)
Abdominal	0 (0%)
**Dropout rate**	0/36 (0%)
**Complication rate**	1/36 (3%)
**Assessed as able to fully comprehend discussion**	36/36 (100%)
**Informed consent obtained with family member present**	36/36 (100%)

**Table 3.** Reason for not pursuing fertility preservation following counselling.

Reason for not pursuing fertility preservation	*N* (%)
Patient/parental choice (Examples of reasons stated: ‘do not wish to be a biological parent’; ‘process of egg freezing is daunting’; ‘idea of coming off testosterone and puberty blockers is daunting’; ‘egg freezing process would be disruptive to education’; ‘do not wish to delay medical treatment’; ‘wish to wait until I’m 18 years old’)	62/102 (61%)
Medically unfit	14/102 (14%)
No funding	9/102 (9%)
Not medically indicated	16/102 (16%)

## Discussion

Fertility preservation forms a fundamental aspect of the management of adolescents who require gonadotoxic treatment, and its remit continues to broaden with ongoing improvements in childhood cancer survival and the growing number of benign conditions requiring gonadotoxic treatment (Oktay *et al.*, 2018; Yasmin *et al.*, 2018; Anderson *et al.*, 2020; Lambertini *et al.*, 2020; Mulder *et al.*, 2021). Whilst there is consensus that clinicians should offer oocyte cryopreservation for fertility preservation in post-menarchal patients at risk of premature ovarian insufficiency, in practice there are several challenges associated with performing fertility preservation in this population. These include assessment of capacity to consent in young adolescents, assessment of medical fitness in individuals who are medically unwell, and decisions surrounding appropriate ovarian stimulation protocols (Oktay *et al.*, 2018; Yasmin *et al.*, 2018; Anderson *et al.*, 2020; Lambertini *et al.*, 2020; Mulder *et al.*, 2021). We share clinical and ethical perspectives gained from performing 36 cycles of ovarian stimulation and oocyte cryopreservation in adolescents, forming one of the largest case series in this population to date. Our study highlights that in the correct setting, oocyte cryopreservation offers a feasible method of fertility preservation in adolescents, with all initiated cycles of oocyte cryopreservation in this cohort proceeding to completion and yielding oocyte numbers according to ovarian reserve.

### Indication for oocyte cryopreservation

Performing a risk assessment of the gonadotoxic impact of a child’s medical condition and treatment is key to counselling children and their families appropriately about the available fertility preservation options, which will depend upon pubertal status and the urgency of initiating therapy (Yasmin *et al.*, 2018; Lambertini *et al.*, 2020). Ovarian tissue cryopreservation is an option for pre-menarchal and post-menarchal children where there is an urgency to commence chemotherapy, radiotherapy, or surgical treatment immediately (Latif *et al.*, 2023). Oocyte cryopreservation is an option for post-menarchal adolescents with 2 weeks available to complete ovarian stimulation and egg collection (Anderson *et al.*, 2020; Mulder *et al.*, 2021). In addition, it offers a treatment option to post-menarchal children in whom ovarian tissue cryopreservation carries the risk of reintroducing malignant cells such as those with leukaemia, providing a realistic option for achieving a future live birth, though there are emerging studies of successful re-implantation of paediatric cryopreserved ovarian tissue (Meirow *et al.*, 2016; Silber *et al.*, 2018; Rodriguez-Wallberg *et al.*, 2021). It also offers an alternative treatment option for those with intermediate risk of gonadotoxicity from treatment, without the associated reduction in ovarian reserve encountered with ovarian tissue cryopreservation, and for those in whom the risks of laparoscopy and its associated recovery are not acceptable (Yasmin *et al.*, 2021).

### Assessment of medical fitness

The most common indication for oocyte cryopreservation in our patient cohort was haematological malignancy (39%, *n* = 14/36), which can be a challenging group as there is often a need to start treatment urgently, and children may be unwell at the time of presentation. Scheduling of ovarian stimulation and egg collection at a time when the child is medically fit is critical to performing egg collection safely, which may require optimization of blood counts through transfusion of red cells or platelets and requires close coordination between the multi-disciplinary team of reproductive medicine clinicians, oncologists, and clinical nurse specialists. An assessment of thrombosis risk is needed, with consideration of low molecular weight heparin use during ovarian stimulation and following egg collection. In children with substantial mediastinal disease where deep sedation is deemed unsafe, a discussion surrounding vaginal egg collection under local anaesthetic can be considered (Yasmin *et al.*, 2021). In the present study, there were no thromboembolic, anaesthetic, or surgical complications encountered during ovarian stimulation or oocyte retrieval.

### Response to ovarian stimulation

The role of standard markers of ovarian reserve, such as AMH and AFC, in predicting response to ovarian stimulation is unclear in the adolescent age group, and standardized monitoring and stimulation protocols have not yet been established in the paediatric and adolescent population (Slonim *et al.*, 2023). Previous studies report discrepancies between ovarian reserve tests and the number of oocytes cryopreserved in teenagers (Lavery *et al.*, 2016; Garg *et al.*, 2019). Our results indicate that response to ovarian stimulation in adolescents is comparable to that in adults with an even spread in oocyte yield across the 13–18 years age group (Fig. 3). Post-menarchal adolescents as young as 13 years achieved an adequate oocyte yield and oocyte maturity rate. We noted a strong correlation between AMH, AFC, and the number of oocytes cryopreserved, indicating AMH and AFC are useful markers of ovarian reserve in adolescents to guide clinicians regarding gonadotrophin dosage for ovarian stimulation (Fig. 2).

In this cohort, all initiated cycles of oocyte cryopreservation proceeded to completion. Oocyte yield and maturity were predictable according to ovarian reserve, and response to ovarian stimulation was comparable to that in adults. Reassuringly, there were no patients with normal ovarian reserve who responded poorly to ovarian stimulation. There were five individuals who had fewer than five oocytes cryopreserved, some of whom had received previous chemotherapy, and oocyte yield was in keeping with their ovarian reserve.

The probability of livebirth is dependent upon the age of the patient and the number of oocytes cryopreserved, with 15 cryopreserved oocytes resulting in approximately a 70% chance of a livebirth (Cobo *et al.*, 2016), although data specific to the adolescent age group are not currently available. The median number of oocytes cryopreserved was 14 in our patient cohort, indicating a realistic prospect of live birth for these individuals. We found dual stimulation increased oocyte yield and was well tolerated (*n* = 4) and therefore could be considered as an approach in adolescents, particularly those with low ovarian reserve, although larger studies are needed to fully assess the burden of undergoing two cycles of oocyte cryopreservation at a young age. Progestin-primed ovarian stimulation offers a valuable alternative to the antagonist protocol as it reduces the burden of treatment due to fewer injections and improves cost-effectiveness (Alabi *et al.*, 2024).

The ideal time to consider fertility preservation is prior to initiation of therapies that may decrease fertility or cause sterility (Anderson *et al.*, 2020); however, this may not always be possible. In this study, those who had received previous chemotherapy (19%, *n* = 7/36) had lower ovarian reserve and required a higher dose of gonadotropins for ovarian stimulation. Nevertheless, all individuals responded to ovarian stimulation and were successful in cryopreserving oocytes.

### Ovarian stimulation in transgender males

Experience in performing ovarian stimulation and oocyte retrieval with concomitant testosterone use is increasing, offering an approach to those who wish to avoid discontinuing testosterone a chance to pursue fertility preservation (Amir *et al.*, 2020; Stark and Mok-Lin, 2022). Stepping down from a long-acting intramuscular testosterone to a short-acting transdermal preparation such as testosterone gel can reduce the time that a person discontinues testosterone (Foo *et al.*, 2024). Transgender males using GnRH analogues may continue these during ovarian stimulation if they wish, alongside counselling regarding OHSS risk and prolonged stimulation (Foo *et al.*, 2024). The aromatase inhibitor Letrozole can be used to suppress estradiol levels to reduce the risk of mental burden from dysphoric triggers in transgender males during ovarian stimulation (Foo *et al.*, 2024).

### Complications

There were no serious complications in our study group. There was one case of mild OHSS in a 17-year-old transgender male who was taking GnRH analogues for pituitary suppression in whom trigger with recombinant HCG was needed, highlighting that in the context of a suppressed HPO axis, AFC and AMH may not be a true reflection of ovarian reserve. Careful dose selection with close monitoring and dose adjustment according to response is needed to reduce the risk of OHSS in those with pituitary suppression.

### Ethical considerations

Ovarian stimulation and oocyte retrieval can be physically and emotionally demanding in the adult population, but the extent of psychological impact has not been studied in the paediatric and adolescent population (McDougall *et al.*, 2018; Logan *et al.*, 2019; Young *et al.*, 2019). In our study, all individuals tolerated ovarian stimulation well and underwent oocyte cryopreservation cycles to completion, including dual stimulation cycles. One 13-year-old patient undergoing oocyte cryopreservation for chronic granulomatous disease awaiting stem cell treatment was offered a second cycle of stimulation to increase oocyte yield but declined. This decision was made following a discussion between the patient, parents, and clinician, in which the rationale for offering a further cycle of treatment and the implications of not pursuing further stimulation were explained in age-appropriate language, and written information was provided. Time was allocated for the family to reflect on this discussion, and an interval follow-up appointment was organized to review their decision, at which time the patient explained that it was her personal choice not to have a further stimulation cycle. However, this patient did wish to receive follow-up once she completed her medical treatment; on review a year following her treatment, she had resumed menses. A limitation of our study is that formal psychological evaluation was not performed, and we recommend this is incorporated in future research studies performed within this population so that the true psychological burden of undergoing oocyte cryopreservation during adolescence can be further evaluated.

Capacity for autonomous decision-making varies greatly among young people, and our study highlights that careful assessment of capacity to consent will enable competent young people to participate in decision-making. Young individuals need to be supported by experienced clinicians and psychologists to develop sufficient understanding to come to a voluntary, well-reasoned, and carefully considered decision to support their personal aspirations for future fertility through a process of shared decision-making (McDougall *et al.*, 2018; Mertes and Pennings, 2022). Clinicians need to discuss the known and potential risks of fertility preservation procedures, the benefits and likely outcomes of alternatives, including not pursuing fertility preservation, and to provide a careful explanation where there is a lack of information about long-term outcomes of fertility preservation procedures. A key ethical consideration is the concern surrounding oocyte quality in the adolescent population. There is a need for clinicians to provide a careful explanation regarding higher reported aneuploidy rates in oocytes of young patients (Gruhn *et al.*, 2019) with sensible counselling regarding uncertainties around live birth rates specific to adolescents since long-term outcome data on live birth rates in this age group are not yet available.

Of equal importance, given the challenging clinical and ethical perspectives, is an analysis and understanding of the 57% who did not proceed with treatment, and this paper is the first to evaluate the significant but unexplored area of why adolescent patients may not proceed with fertility preservation (Table 3). Reasons stated for not proceeding with fertility preservation included that they ‘did not wish to be a biological parent’, that ‘the process of egg freezing was daunting’, that ‘the idea of coming off testosterone and puberty blockers was daunting’, that ‘the egg freezing process would be disruptive to their education’ and that ‘they wished to wait till they were 18 years old’ (Table 3). Importantly, clinicians need to consider the timing of fertility counselling in adolescents for whom genetic parenthood may not be an immediate priority and that biological parenthood may not be desired.

Nevertheless, uptake rates within our cohort for performing oocyte cryopreservation in those undergoing gender reassignment were slightly higher than previously published studies, albeit our patient cohort size was substantially smaller (Insogna *et al.*, 2020; Amir *et al*., 2020; Alpern *et al.*, 2022). The availability of NHS funding may have contributed to increased uptake in our study. In line with our local practice, both parents and children were counselled, and we did not encounter any conflicts between a child’s and parents’ wishes in our cohort, possibly a reflection of the counselling which was delivered by clinicians who have experience in the field of paediatric and adolescent gynaecology and are trained to facilitate decision-making in young individuals.

### Barriers to provision

While there is international consensus that fertility preservation services need to be provided to children at risk of infertility, variation in the availability of fertility preservation services and the provision of public sector funding for children exists across the UK and Europe (Anderson *et al.*, 2020; Newton *et al.*, 2022; Latif *et al.*, 2023). Provision of fertility preservation services in children requires specialized training of healthcare personnel and dedicated resources; however, many fertility clinics lack the expertise or resources to manage paediatric cases effectively, and therefore it will not be possible to apply our study findings in all settings. Establishing relationships with specialist centres that have expertise to counsel and treat children is crucial for improving healthcare services for young patients who require fertility preservation. Centralized funding is one approach to support the equitable provision of fertility preservation services.

Counselling young patients and their families about their options for fertility preservation is complex, and long-term follow-up studies of efficacy, safety, and utilization of oocyte cryopreservation in the adolescent population are needed with data on livebirth outcomes in particular so that clinicians are able to counsel patients appropriately (Anderson *et al.*, 2015; Garg *et al.*, 2019; Slonim *et al.*, 2023). Our study is limited by its retrospective design, relatively small sample size, and that there are no patients in our study cohort who have yet returned to use their gametes. Higher rates of foetal aneuploidy have been described in adolescent pregnancy when compared with women in their twenties (Franasiak *et al.*, 2014), and the future ability to attain a viable pregnancy and live birth is not guaranteed, with current literature consisting of a single case report of a livebirth from cryopreserved oocytes achieved in a 17-year-old assigned female at birth (Kim and Hong, 2011).

There are often concerns about acceptability of transvaginal procedures in children, even though there is lower surgical risk compared with transabdominal egg collection (Klipstein *et al.*, 2020). Our cohort included children from a diverse range of ethnicities, all of whom agreed to have transvaginal egg collection. Our study highlights that appropriate counselling assists in achieving good compliance with transvaginal egg collection.

## Conclusion

Our study shows that oocyte cryopreservation offers a viable form of fertility preservation in early adolescents with appropriate counselling, and that response to ovarian stimulation in adolescents is predictable and comparable to that in adults. Clinician experience, the correct setting, and provision of funding will enable a permissive environment for performing oocyte cryopreservation in adolescents. Studies expanding on our findings, particularly with data on livebirth outcomes, are needed to support clinicians to counsel patients and perform oocyte cryopreservation in adolescents.

## Data Availability

The data underlying this article will be shared on reasonable request to the corresponding author.
